# Requirements for essential micronutrients during caloric restriction and fasting

**DOI:** 10.3389/fnut.2024.1363181

**Published:** 2024-02-28

**Authors:** Weiguo Zhang, Peng Chen, Shaofeng Huo, Xiaomin Huang, Youyou Zhao

**Affiliations:** ^1^Las Colinas Institutes, Irving, TX, United States; ^2^Sirio Pharma, R&D, Shantou, Guangdong, China; ^3^Sirio Pharma, R&D, Shanghai, China

**Keywords:** caloric restriction, micronutrients (vitamins and minerals), obesity, aging, non-human primates, metabolism

## Abstract

Caloric restriction (CR) or energy restriction, when carefully designed, monitored, and implemented in self-motivated and compliant individuals, proves to be a viable non-pharmacologic strategy for human weight control and obesity management. Beyond its role in weight management, CR has the potential to impede responses involved not only in the pathogenesis of various diseases but also in the aging process in adults, thereby being proposed to promote a healthier and longer life. The core objective of implementing caloric restriction is to establish a balance between energy intake and expenditure, typically involving a reduction in intake and an increase in expenditure—a negative balance at least initially. It may transition toward and maintain a more desired equilibrium over time. However, it is essential to note that CR may lead to a proportional reduction in micronutrient intake unless corresponding supplementation is provided. Historical human case reports on CR have consistently maintained adequate intakes (AI) or recommended dietary allowances (RDA) for essential micronutrients, including vitamins and minerals. Similarly, longevity studies involving non-human primates have upheld micronutrient consumption levels comparable to control groups or baseline measures. Recent randomized controlled trials (RCTs) have also endorsed daily supplementation of multivitamins and minerals to meet micronutrient needs. However, aside from these human case reports, limited human trials, and primate experiments, there remains a notable gap in human research specifically addressing precise micronutrient requirements during CR. While adhering to AI or RDA for minerals and vitamins appears sensible in the current practice, it’s important to recognize that these guidelines are formulated for generally healthy populations under standard circumstances. The adequacy of these guidelines in the setting of prolonged and profound negative energy balance remains unclear. From perspectives of evidence-based medicine and precision nutrition, this field necessitates comprehensive exploration to uncover the intricacies of absorption, utilization, and metabolism and the requirement of each hydrophilic and lipophilic vitamin and mineral during these special periods. Such investigations are crucial to determine whether existing daily dietary recommendations for micronutrients are quantitatively inadequate, excessive, or appropriate when energy balance remains negative over extended durations.

*“The fundamental things apply as time goes by.”*

*— Herman Hupfeld*

## 1 Introduction

Energy metabolism is a vital physiological and biochemical process that involves two important parts: 1. the energy intake from food and beverages; and 2. the energy expenditure for maintaining a basal metabolic rate, conducting thermogenesis, and performing physical activities (1). The human body can and needs to store the surplus of energy ingested and absorbed—an inheritable trait believed to survive life-threatening challenges like starvation and energy deficit, which have not been rare in the history of human evolution. The imbalance between energy intake and energy expenditure causes a variety of health disorders and diseases. Just from simple anthropometric measurements, for example, there can be wasting or stunting on one hand and overweight or obesity on the other hand.

Caloric restriction (CR) or energy restriction, known as low-calorie diets and beverages, is generally defined as consuming 20 to 40% fewer calories than normal. CR (including fasting in this article) has been the conventional approach to lifestyle modification for several reasons. First, obesity has rapidly become an epidemic in the last century, affecting almost all ages and socioeconomic groups in developed and developing countries. As reported by the WHO in 2021, approximately 2.2 billion men and women are overweight or obese, representing more than 40% of the global population (Accessed on Feb 15, 2022).^1^ By aiming to reduce energy intake and utilizing more tissue fat (and maybe protein to some extent) to fuel the machinery of the body, CR constitutes one of the non-invasive and non-pharmacologic management methods for overweight and obese populations. Second, inspired by the benefit of CR observed in other experimental paradigms, many are convinced that practicing CR in early adulthood and midlife constitutes a healthy lifestyle, minimizing the risk of age-related diseases (2), particularly those like cardiovascular, metabolic, and cognitive disorders that are commonly accompanied by unrestricted calorie intake. Without calorie surplus, the human lifespan is supposed to be potentiated. Third, the approach is natural and non-invasive, inexpensive, self-implementable, and effective.

Nevertheless, to catabolize fat tissue or prevent fat from accumulation during negative energy balance (i.e., when energy expenditure exceeds intake) is primarily what the CR aims directly at, which constitutes one of the main bodyweight loss or control regimens for obesity itself and beyond, e.g., blood pressure and heart rate reduction (3), diabetes remission (4), relief of epileptic seizures (5), cancer prevention (6); and more interestingly, for promoting healthy aging or a longer and healthier lifespan (7). In the literature and current practice, negative energy balance can be achieved through various fasting patterns from partial to zero energy (food) intake, representing an extreme scenario of deprivation of all macro-or micro-nutrients regardless of whether they are energy-generating or not.

A fundamental unanswered question is, “When all or some of the macronutrients (i.e., foods) are limited or deprived intermittently or continuously for a prolonged period, what is the requirement of the human body for micronutrients that are parts of various foods?” This may be affected by multiple factors but should be largely determined by the state of negative energy balance, in which biochemical and biophysical processes are altered in the body, as are the pharmacokinetics and pharmacodynamics of the micronutrients.

This brief review aims to underscore the important findings about micronutrient requirements during CR with a specific focus on insights gleaned from both non-human primates and human subjects. From these reports, and the perspective of evidence-based nutrition or precision medicine, we try to elucidate both the known and unknown aspects, thus identifying areas that warrant further scientific investigation. Moreover, it is noteworthy that the writing and submission of this manuscript coincides with the 50th anniversary of the publication of “Features of a successful therapeutic fast of 382 days duration” (8) (see detailed discussion later), prompting reflection on the interesting progress made since then in the field.

## 2 Micronutrients in caloric restriction in non-human primates

Among mammals, non-human primates share a remarkable genetic similarity of over 90% with the human genome (9), positioning them as valuable models for simulating the aging process in humans. Several long-term monkey studies have investigated the effects of extended CR on cardiovascular disease, cancer, and longevity in captive primates (10–15). Understanding how these close relatives of humans were supplemented with micronutrients during CR is therefore very relevant to the focus of the current review.

Crab-eating macaque (*Macaca fascicularis*) study (Wake Forest University). Based on their total plasma cholesterol to high-density lipoprotein cholesterol (HDLC) ratio, age, and body weight, a total of 32 cynomolgus monkeys were randomly divided into a control group and a CR group with a 30% reduction in calorie intake. To compensate for the approximately 30% reduction in vitamin intake due to CR, additional vitamins (and minerals) were incorporated into the CR diet (i.e., the diet formulation contained 2.50 g complete vitamin mix per 100 g for control monkeys and 3.57 g for the CR group), rendering the vitamin intake comparable between the two groups (Table 1). This adjustment ensured a consistent provision of the same quantity of these micronutrients throughout the study (11). Over 4 years, a 30% CR led to improvements in insulin sensitivity and reductions in both body weight and intraabdominal fat with aging.

**Table 1:** Diet compositions of four major non-human primate studies.

	Micronutrients vitamins and minerals	Macronutrients carbohydrate%	Protein%	Fat%	Feeding arrangement
Wake Forest University^1^					
Control Group	2.50 g/100 g diet	49.7	20.5	29.8	Ad libitum (100 calories/kg body weight)
CR Group	3.57 g/100 g diet	49.7	20.5	29.8	
CR Group approx. Intake	2.50 g/100 g diet				30% reduction from baseline
University of Maryland^2^					
Control Group	Chewable multiple vitamins	70	17	13	Ad libitum
CR Group	Chewable multiple vitamins				
CR Group Intake	Chewable multiple vitamins				Titrated to keep body weight 10–11 kg
University of Wisconsin^3^					
Control Group	100% RDA	60.9	17.3	5.0	Ad libitum daytime, no food overnight
CR Group	130% RDA	56.9	13.1	10.6	
CR Group approx. Intake	91% RDA				30% reduction from baseline
National Institute on Aging^3^					
Control Group	140% RDA	56.9	17.3	5.0	Ad libitum with slight restriction
CR Group	140% RDA	56.9	17.3	5.0	
CR Group approx. Intake	98% RDA				30% reduction from matched control

The actual intake of micronutrients (i.e., vitamins) in monkeys with caloric restriction is maintained approximately the same as the level taken by control animals (i.e., ad libitum) (11–15).

1 The quantitative breakdown of individual micronutrients was not indicated in the original publication (11).

2 The components of chewable multiple vitamins were not indicated in the original publication (12).

3 For detailed information on individual micronutrients, see National Research Council publications (16, 17).

Rhesus macaque (*Macaca mulatta*) study (University of Maryland). A total of 117 rhesus monkeys were observed over 25 years, among which, 8 were subjected to dietary restriction (DR) while 109 were maintained under *ad libitum* feeding conditions. In this study, the DR was to maintain a body weight of 10 to 11 kg (a range of normal lean adult monkeys) through individualized titrating of monkey chow allotments. When compared to the 8 monkeys with DR, the 58 healthy monkeys with *ad libitum* feeding displayed a 2.6-fold increase in mortality risk. Additionally, among the 33 monkeys with *ad libitum* feeding and concurrent hyperinsulinemia, there was a 3.7-fold increase in mortality (12). It is noteworthy that both groups received a daily regimen of chewable multivitamins (Table 1).

Rhesus macaque (*Macaca mulatta*) study (Wisconsin National Primate Research Center [WNPRC], University of Wisconsin). The WNPRC study was launched in 1989 and enrolled a cohort of 76 adult monkeys (averaging around 8 years of age). These monkeys were matched and randomized into either a control group or a CR group (*n* = 38 each). The CR approach involved a stepwise decrease of 10% of an individual’s baseline food intake per month over 3 months, ultimately achieving a desired 30% restriction. A few decades later, the research unveiled the reductions of not only body weight and fat mass, but also the incidence of several age-related diseases, including cardiovascular disease, cancer, diabetes, sarcopenia, and brain atrophy (13). Fascinatingly, CR helped reduce age-related mortality and all-cause mortality, leading to distinct survival curves that emerged and diverged further over time. Like in the NIA study, the 30% reduction in food intake would normally lead to proportional reductions in the intake of vitamins and minerals. To address this, the diet for the CR group was fortified to a level that was 130% of RDA (Table 1), so that the monkeys in the intervention group received an equivalent amount of micronutrients as their control counterparts did (13, 14).

Rhesus macaque (*Macaca mulatta*) study (National Institute on Aging [NIA]). Initiated in 1987, the NIA study involved 121 monkeys of different ages. Within this cohort, 62 monkeys adhered to a control diet, while 57 underwent CR, experiencing a 30% reduction in their diet compared to controls matched for height, age, and sex. After almost three decades, the intervention was found to have improved the general metabolic profile, i.e., body weight, serum triglycerides, cholesterol, and glucose. However, despite these improvements, the intervention did not improve the survival curves for both the young and older monkeys. Nevertheless, among young monkeys, the oxidative stress indicated by lower levels of free isoprostanes (derived from free radical-catalyzed peroxidation of fatty acids) in plasma was reduced. Additionally, a discernible delay in the incidence of cardiovascular disease, cancer, diabetes, arthritis, and diverticulosis was observed but did not reach statistical significance (*p* = 0.06). Although the monkeys on dietary intervention consumed 30% less food, the intakes of vitamins and minerals were not proportionally reduced. Rather, the diet for the CR monkeys was supplemented with an additional 40% of the recommended dietary allowance (RDA) of vitamins and minerals (Table 1), which ensured that each individual, irrespective of whether they received the intervention, met the RDA for essential micronutrients (15).

Regarding micronutrient intakes, the UW study fed two different diets and only the CR monkeys were supplemented (15, 18); however, the NIA study used one diet for both CR and control monkeys, which was supplemented with an additional 40% of the daily-recommended allowance. Thus, the NIA diet formulation super-supplemented the control monkeys. It is important to point out that in both the NIA and UW studies, CR was implemented so that control monkeys were no longer fed *ad libitum*. The food allocations were determined based on published data from the National Research Council to provide approximate *ad libitum* intake based on age and body weight of mature control monkeys without overfeeding (16). The latest allotments were published in 2003 (17), and an exact quantitative breakdown of individual micronutrients was provided.

There were other studies involving squirrel monkeys (*Saimiri Sciureus* and *Saimiri Boliviensis*) and baboon monkeys (Papio spp.) (19–21), which have been discussed elsewhere (22) but are not included in this review because of their relevance (e.g., research aim, sample size, study duration, etc.) to the present writing framework. Collectively, CR studies conducted on monkeys aimed to thwart the age-related onset of non-communicable diseases and extend their lifespan, mirroring outcomes observed in other species (e.g., mice, rats, and dogs) (23–25). The investigators of the NIA study and the UW study jointly concluded that “health benefits of CR are conserved in monkeys” and suggested that “CR mechanisms are likely translatable to human health” (18). the research in long-lived monkeys has bridged some gaps between experimental paradigms in short-lived animals and human beings, contributing to a more comprehensive understanding of the potential health benefits of CR across species.

## 3 Micronutrients in caloric restriction in obese humans

CR to treat obesity was prompted in the first half of the 20th century, if not earlier. This coincided with the progress in understanding the physiological roles of micronutrients (such as vitamins) in health and disease processes. Vitamins were chemically isolated, biologically described, synthesized, and on the verge of being commercially available. The essentiality of micronutrients was considered in the early CR studies. For instance, in a report in 1931, a total of 187 obese patients underwent low-calorie diets that provided 400–600 calories daily for a duration of 8.7 weeks. This resulted in an average body weight loss of 30 pounds (13.6 Kg) at the end, and the patients were kept in nitrogen equilibrium without acidosis and without depriving inorganic salts and vitamins. The latter was because when the study was designed, the investigators had the assumption that “human fat, as other animal fat, [sic] stored the fat-soluble vitamins was considered reasonable at first. It has been found [sic] desirable to employ supplementary daily doses of viosterol and occasionally whole milk. The remaining vitamins were adequately supplied by the carbohydrate ration in which the 5 percent vegetables were largely used together with small quantities of growing yeast, orange juice and milk” (26).

Some later publications reported cases of total abstention from taking food (27) and intermittent fasting in obese subjects (28). One of the very interesting cases in the literature is that of an obese 27-year-old outpatient who weighed 207 kg (456 lbs.) on admission; they underwent fasting for 382 days and weighed 82 kg (180 lbs.) at the end of the fasting period (8). Such prolonged starvation could have hazardous consequences or cause mortality; the patient was, however, observed to have been without overt illness despite losing 125 kg (276 lbs.). It must also be emphasized that the patient took multivitamins and mineral supplements daily (i.e., ‘Multivite’ manufactured by BDH, vitamin C, and yeast for the first 10 months and then ‘Paladac’, manufactured by Parke Davis, for the remaining 3 months). The patient only regained approximately 10% of the weight in the 5 years afterward.

Micronutrient deficiency is more prevalent in the obese population than in those with normal body weight (29, 30). In a study of 104 obese individuals with an average body mass index of 40.9, the intakes of retinol, ß-carotene, vitamin D, folate, iron, and iodine were below Dietary Reference Intakes (DRI) in >75% of the study population, and insufficient intakes of vitamin E, C, and calcium were identified in >50% of the same population. Of these subjects, 32 underwent a low-calorie formula diet that provided 100% of the daily micronutrient requirements according to DRI and lost approximately 20 kg body weight after 3 months. However, 100% of micronutrients did not correct all profiles of the micronutrient deficiency; for instance, serum vitamin C, iron, and calcium were not improved (31).

Although a handful of studies did not supplement micronutrients during CR, among which some focused specifically on the changes in vitamins in circulation (32, 33), micronutrient supplementations are the mainstay in the research (34–36) and have been endorsed by authoritative organizations (to be discussed in section 4).

## 4 Micronutrients in caloric restriction in non-obese humans

As mentioned previously, CR has been used as an anti-aging approach by others who are unnecessarily obese and are motivated by health, well-being, and longevity. Some clinical studies have reported short-term CR involving undereating in non-obese human subjects. These studies were not directly intended to offer obesity therapy per se, and the duration varied from weeks to months. The reduction of energy intake was often mild, so the status of micronutrients was often overlooked in the study design and outcome assessment (37–39), particularly in observational and short-term studies. In the latest RCT studies, micronutrient supplements were often adequately addressed.

Non-RCT studies. Minnesota Starvation Experiment was a longer study conducted on young lean subjects as early as in the 1940s. After a control period of 12 weeks (receiving 3,200 kcal daily), they underwent semi-starvation by consuming 1800 kcal and walking 5 km daily for 24 weeks, resulting in an average weight loss of 25% (40). The diet consisted of potatoes, cabbage, radishes, and grains, with only a few grams of animal protein added per week, and was deficient in both macronutrients and micronutrients (e.g., insufficient intake of protein, vegetables, and fruit), as the study was designed to simulate cachexia and malnutrition that was experienced during World War II and learn about the biochemistry, physiology, and psychology of wartime diet and famine (41, 42). In this study, reduced aerobic capacity, chronic weakness, and lower limb edema, as well as abnormal psychological behaviors (i.e., emotional distress, confusion, apathy, depression, hysteria, hypochondriasis, suicidal thoughts, and loss of sex drive) were observed (43), which should have been directly related to malnutrition and mental stress, among other causes. These abnormalities were reversible. Interestingly, 19 of the 36 original participants were still alive 60 years later and 18 of them, who participated in an interview from July 2003 to February 2004, were in their 80s (41). In terms of life expectancy, 50% of the original participants lived 8 years longer than men born in 1920 (43). This was a 6-month CR study without any micronutrient supplementation. It can be postulated that a 44% reduction in energy intake might have been accompanied by a proportional reduction in micronutrients during this period. The question is whether these subjects were better off with an adequate intake of micronutrients from either vegetables and fruits or dietary supplements.

In 1968, a study reported the effects of short-term CR on mineral balance and vitamin excretion (44). Eight young men first consumed a control diet that provided 3,230 kcal per day for a week. This was followed by consuming 420 kcal of carbohydrates daily for a period of 10 days, while half of them received mineral supplementation and the other half did not. It was found that electrolyte excretion in urine was greatly decreased during the first 3 days, reflecting the rapid adaptation to zero intake of minerals; losses of sodium and potassium were greatest during the first 2 days of restriction and continued to decrease during this phase, while daily calcium and magnesium losses increased as restriction progressed. With mineral supplementation, the mineral balances for sodium, calcium, and magnesium were maintained. Although the supplementation reduced potassium excretion, it did not normalize its balance (44). Urinary excretion of thiamine, riboflavin, niacin, and vitamin B6 was significantly reduced during CR when no vitamins were ingested, which was in agreement with an earlier report (45). Like minerals, vitamin excretion should largely reflect the rapid adaptation to the depleted intake.

Some earlier epidemiology studies did not observe striking diseases due to starvation-caused vitamin deficiency. For example, children in Holland during World War II had low food rations (the energy intake averaged 800 calories, protein averaged 25 g, fat averaged 12 g daily, and vitamin values were low) for 7 months, but no diseases related to vitamin deficiency were found other than vitamin A deficiency-induced hyperkeratosis. However, mineral deficiency-related anemia and decalcification of the bones were prevalent (46). In 2022, the results of a 10-day fasting study were reported. The study aimed to evaluate the impact of vitamins and other metabolic homeostasis, so no micronutrients were provided to 13 healthy male participants. It was found that accompanying 9.8% (7.28 kg) body weight loss, lipid-soluble vitamins (i.e., A, D, E) were elevated in circulation, whereas water-soluble vitamins (i.e., B1, B2, B6, B9, B12, and C) were largely unchanged (47). The short-term trend of the lipid-soluble vitamins is in accordance with a previous assumption (26).

Taken together, some studies did not conclude the necessity of micronutrient supplements during CR (44, 47, 48). The studies are largely observational, are almost all short-term, and involve a limited number of healthy subjects. The absence of micronutrient deficiency or overt clinical symptoms of micronutrient deficiency during the study periods does not mean that (1) deficiency would not occur if the study period lasted longer, and (2) the deficiency had no long-term impact on health compared with those who were not deficient; the latter should serve as controls but were not included in the design of the study. As such, participants in later studies were still provided with micronutrients (49–51).

RCT studies. There are a handful of trials available with RCT designs (52–55), of which larger-scale trials like the Comprehensive Assessment of Long-Term Effects of Reducing Intake of Energy (i.e., CALERIE) study are highlighted in the field because of their randomized and controlled design, large scale, and longer duration of intervention. Following CALERIE phase 1, which tested the feasibility and effects of CR of 10 to 30% for 6 to 12 months, CALERIE phase 2 was a comprehensive RCT conducted in 218 young and middle-aged healthy men (21–50 years) and women (21–47 years), who were not obese (BMI 22·0–27·9 kg/m2), and received, on average, an 11.9% lower calorie intake for a period of 24 months (56).

Compared with the *ad libitum* arm as control, the remarkable change in the intervention arm was the reduction in body weight, fat mass, and trunk mass, and the increase in the lean-to-fat mass ratio. The improvement in body composition was associated with improvements in cardiovascular risk markers (e.g., reductions of fasting insulin, total cholesterol, LDL-C, and triglycerides; and HDL-C), metabolic and endocrinal function (e.g., reduction of leptin and fasting insulin, and an increase of adiponectin and glucose tolerance), inflammation, oxidative stress, and immune function (e.g., reduction of TNF-alpha, C reactive protein) and mental, psychological, skeletomuscular, and behavioral outcome measures (56–58).

Amazingly, analysis with the Klemera–Doubal method on biomarkers from the CALERIE biobank revealed that biological aging was at a pace of 0.71 years per 12-month period in the control group. The aging pace was decelerated to 0.11 ‘years’ per 12-month period in the CR group (59).

Likewise, in the absence of any adverse effects between the intervention group and the control group, one of the prerequisites to ensure the safety and benefits of CR may be daily supplementation with multivitamins and minerals (Nature Made Multi Complete, Mission Hills, CA, USA) as well as an additional calcium supplement (1000 mg/d, Douglas Laboratories, Pittsburgh, PA, USA) (60) to the participants, which had been previously tested in the previous pilot or other similar studies (52, 53, 61), thus meeting the national dietary guidelines (62).

## 5 Micronutrient requirements during calorie restriction

Dietary Reference Intakes. The DRIs for each micronutrient consist of Estimated Average Requirements (EAR), Recommended Dietary Allowance (RDA), Adequate Intake (AI), and Tolerable Upper Intake Level (UL). In a specific life stage/age and gender group, EAR should meet the needs of 50% of healthy individuals, and RDA, calculated from EAR, should meet 97.5% (63). Overall, “DRIs are intended for application to the apparently healthy population rather than those with medical conditions requiring specialized diets” (64). Deficient and insufficient intake of micronutrients often occurs with a deficit in food (energy) intake or due to consuming energy-dense but micronutrient-poor diets, referring to the concept of hidden hunger.

It is almost certain that reduced calorie intake is often correlated to reduced micronutrient intake proportionally (65). It is largely unknown what the exact requirement for each micronutrient is during CR, particularly over a very prolonged timeframe. In each given group of participants, the degree of micronutrient deficiency may depend on different patterns of CR, e.g., intermittent, periodic, continuous fasting, and the stage of the fasting, which may determine the pharmacokinetics and pharmacodynamics of micronutrients in that circumstance.

Undogmatic examples of micronutrient dynamics. One example is vitamin D. It has been found that in CALERIE 2, serum 25OHD concentrations significantly increased in the CR group compared with *ad libitum* at 12 months and 24 months, unaccompanied by changes in PTH. An earlier report from a 12-month trial found that the higher the weight loss during CR, the higher the increase in serum 25OHD (33). One explanation is the sequestration of fat-soluble substances that are released from adipose tissue. Despite that, however, the loss in bone mineral density (BMD) was reported at approximately 2% during CR at clinically prevalent sites of osteoporotic fractures (60, 66). Some dietary intervention may affect the active hormone 1,25-(OH)2D3 through modulating cytochrome P450 27B1 (CYP27B1) expression in the kidney, leading to an increase in circulating 25OHD and a decline in 1,25-(OH)2D3 (67). As neither 1,25-(OH)2D3 nor CYP27B1 was measured, the precise mechanism is unknown. Nonetheless, in addition to the modulation of related gene expression, reduced BMD might have been caused by reduced bone load as a result of body weight loss (68, 69).

Another example is B vitamins. In obese children and adolescents who received physical training consisting of different activities performed 3 times per day and were assigned to a mixed diet of 908 to 1,195 Cal/day (3,800 to 5,000 kJ/day) with a daily intake of 230 μg, 4 μg, and 1.5 mg for folate, B12, and B6 respectively, decreases were identified in BMI, fat mass (FM), percentage fat mass, insulin, and C-peptide; plasma vitamin B12 was surprisingly increased (from 461 ± 119 to 560 ± 216 pg./mL); and folate remained unchanged during the 3-week weight reduction course (70). In a fasting study lasting 10 days, there was a rapid depletion in vitamin B1 and B6 storage in the body because their urinary excretions were in the low-to-deficient ranges. The B1 and B6 depletion was followed by a decrease in B2 (71).

As seen in a 3-month study, 100% supplementation of micronutrients to obese people receiving a low-calorie formula diet did not correct all serum profiles of micronutrient deficiency (31), which is prevalent in obese populations (72).

Positions of professional authorities. Micronutrient supplementations are suggested by several governmental and professional authorities during CR or similar scenarios. Here are a few examples. Regarding very low-calorie diets (VLCD), the National Task Force on the Prevention and Treatment of Obesity stated, “The caloric levels of VLCDs allowed for the ingestion of essential macronutrients, vitamins, and minerals and avoidance of the losses of lean body mass and severe side effects associated with fasting” and “current VLCD formulations usually contain appropriate vitamins and minerals, making serious abnormalities unusual. Very low-calorie diets using food sources (e.g., lean meat, fish, fowl) will require additional supplementation” (73). For the very low-calorie ketogenic diet (VLCKD) that significantly reduces carbohydrates (50 g/day), the Italian Society of Endocrinology reinforces that “patients on VLCKD must be closely and periodically monitored through physical examination (anthropometric measurements, blood pressure, heart rate, etc.) and laboratory analysis, to prevent dehydration and vitamin/electrolyte abnormalities, which are potentially due to urinary excretion of ketone bodies and poor intake of micronutrients. Hence, proper water intake (at least 2 L of sugarless fluids daily), vitamin/electrolyte and omega-3 polyunsaturated fatty acids supplementation are mandatory, especially during the first phases” (74). The Obesity Management Task Force (OMTF) of the European Association for the Study of Obesity (EASO) states that “supplementations with micronutrients (vitamins and minerals, such as K, Na, Mg, Ca, and omega-3 fatty acids) are suggested” at the active stage of VLCKD, which lasts 8–12 weeks until the subjects lose most of the weight loss target (about 80%) (75). In terms of the anti-aging approach, CR Society International opines that “the goal of Calorie Restriction is to achieve a longer and healthier life by eating fewer calories and consuming adequate vitamins, minerals, and other essential nutrients” (see [CR Society International].^2^ Accessed on Oct 30, 2022).

## 6 Concluding remarks

The exploration of micronutrient requirements during CR is a crucial facet of evidence-based or precision medicine and nutrition. The intricate interplay between energy metabolism, dietary intake, and the demands of the human body during CR necessitates a comprehensive understanding of micronutrient dynamics. Analysis of both non-human primate studies and investigations involving human participants, both obese and non-obese, reveals that CR has transformative potential for weight management, physical and mental health, and longevity.

While some studies raise questions about the adequacy of dietary sources and the potential role of supplements in addressing challenges posed by reduced calorie intake, recent advancements in research, exemplified by the CALERIE studies in which micronutrients were supplemented, offer optimism regarding the benefits of CR on various health biomarkers. Although the cautious approach advocated in the conclusion underscores the current uncertainty surrounding micronutrient requirements during CR, in general, however, the strategies for micronutrient supplementation in these trials are of practical importance (Table 2). Adhering to existing Recommended Dietary Allowances (RDAs) or Adequate Intake (AI) represents a justifiable measure to prevent micronutrient deficiency (hidden hunger) and safeguard nutritional well-being until further experimental results illuminate the intricacies of absorption, utilization, and metabolism during CR.

**Table 2:** Summary of the major advantages of caloric restriction in both nonhuman primates and humans, alongside the risks of micronutrient deficiencies resulting from such restriction.

	Potential benefits	Potential detriments
Short term	Body weight loss Improved insulin resistance Improved glucose tolerance Improved lipid profile Improved inflammation profile...	When CR is applied, the reduced dietary intake often leads to micronutrient insufficiency or deficiency (referred to as hidden hunger), giving rise to various subclinical and clinical manifestations
Long term	Lifestyle and epigenetic modifications via caloric restriction may mitigate the risk of aging-related diseases, promoting a longer and healthier lifespan	The advantages of caloric restriction (CR), arising from lifestyle and epigenetic adjustments, may be compromised over the long term due to micronutrient deficiencies

In essence, this discourse delves into the intricate relationship between caloric restriction, micronutrients, and overall health. Navigating the landscape of CR theories and practices, from obesity management to potential anti-aging interventions, underscores the importance of a comprehensive understanding of micronutrient dynamics. The journey to unravel the unknowns in this domain persists, with a commitment to evidence-based approaches and a thorough consideration of micronutrient needs in pursuing healthier and longer lives.

## Author contributions

WZ: Conceptualization, Data curation, Formal analysis, Writing – original draft, Writing – review & editing. PC: Data curation, Validation, Writing – review & editing. SH: Data curation, Validation, Writing – review & editing. XH: Data curation, Validation, Writing – review & editing. YZ: Conceptualization, Supervision, Validation, Writing – review & editing.

## Footnotes

^1^ https://www.who.int/news/item/07-12-2021-who-accelerates-work-on-nutrition-targets-with-new-commitments

^2^ https://www.crsociety.org/resources/getting_started/
